# News and Notes

**Published:** 1903-09

**Authors:** 


### NEWS AND NOTES.

BARNES MEDICAL COLLEGE, St. Louis, has established pharmaceutical and dental departments.

SIR FREDERICK TREVES will retire from active practice, it is announced in the English newspapers.

BECAUSE of the wetness of the season, there is an enormous increase of mosquitoes along the North Atlantic coast.

THE Swedish Medical Association has lately been reorganized on the same plan as the American Medical Association.

LATE information from Honolulu states that dengue fever continues to prevail in Honolulu and in other districts of the islands.

ISOLATED cases of bubonic plague have occurred at Valparaiso and other towns in Chile. The authorities are taking the strictest precautions.

THE United States Minister at Santiago de Chile has sent a cable despatch to the State Department reporting a serious state of affairs in Chile in consequence of the rapid progress of bubonic plague in nearly all the seaport towns.

THE Sixth International Congress of Psychology, which was to have met in Rome in the autumn of 1904, will be postponed to the spring of 1905 to avoid conflict with the Sixth International Congress of Physiology, which meets at Brussels in the autumn of 1904.

THE late Prof. Carl Gerhardt, of Berlin, would have been seventy years old if he had lived till May 5. On that date a memorial ceremony was held at the Berlin Charité, addressed by Professor Martius, of Rostock. A portrait of Gerhardt was presented by his assistants, to be placed in the medical clinic formerly under his charge.—*Exchange.*

---

ONE of the oldest practicing physicians in the West, Dr. Robert Boal, died at his home in Lacon, Ill., on June 12, at the age of ninety-six. Dr. Boal was prominent in the Illinois legislature long before the civil war and was a stanch supporter of Lincoln. He was one of the organizers of the Illinois Medical Society seventy years ago, and was an intimate friend of Lincoln, Douglas, Baker, and Hardin, and wielded a large power in shaping the affairs of the State of Illinois in the early days.

---

IT is reported, says the *Medical Record,* that the Portuguese government has received a proposal from Prince Francis Henry of Hohenlohe for the formation of a large company for the exclusive establishment and exploitation of sanatoria for the cure of consumption in Madeira. The proposal guarantees that one third of the receipts shall be paid to the State, and that a forfeit of $100,-000 shall be deposited with the Portuguese government. Two sanatoria are to be built within the space of two years.

---

DR. WILEY, of Washington, D. C., who has for some time past conducted experiments in the administration of borax in the preparation of food, with a view to determining its qualities as a harmless or deleterious substance in diet or in the preservation of food, has, according to the *New York Medical Journal,* closed his laboratory and free boarding-house for the summer, one reason assigned being that the proximity of the scientific kitchen to the laboratory is likely to render the temperature of the latter more torrid than desirable. Dr. Wiley expects to resume his experiments on October 1.

---

A MAJOR and surgeon of the army stationed in the Philippines writes that recently, when the chief nurse of a small base hospital in Southern Luzon was sent away, there was a great struggle among the five nurses remaining for the vacant position, which meant a distinct increase in pay. Each one of the five came to the office of the surgeon in charge to show cause why she should be appointed chief nurse, and why none of the others were entitled to that distinction. The young Solomon in charge was "up against it," but gave the following decision: "Each one of you must write on a piece of paper her exact age, and send it sealed to me. The oldest woman will be made chief nurse." There is still a vacancy as chief nurse in a small base hospital in Southern Luzon.

---

ACCORDING to the police of Philadelphia it is not an unusual thing for boys to become intoxicated by inhaling the fumes from kerosene. The vicinity where these debauches have been observed is usually in the neighborhood of the railroad yards where the empty oil cars are stationed. The method of obtaining the fumes is for the boys to climb upon the tank car, place their noses over the manhole, and thus inhale the fumes. The effects produced are similar to those caused by alcohol; first a feeling of exhilaration, then a period of stupor, and following is the period of deep sleep. It is stated that in several instances boys drunk from these fumes have been taken to hospitals in the vicinity. From the meager amount of observation in such cases it is believed that the effects on the system are similar to those produced by alcohol.—*American Medical Journal.*

